# Recent advances in tendon redox biology: the interplay of oxidative stress, calcium signaling, and antioxidant defence mechanisms

**DOI:** 10.3389/fphar.2026.1752491

**Published:** 2026-04-02

**Authors:** Yejiang Tang, Ahmad Alhaskawi, Bing Ruan, Zhenli Yuan

**Affiliations:** 1 Department of Emergency, Zhuji People’s Hospital, Zhuji, Zhejiang, China; 2 Department of Orthopedics, The First Affiliated Hospital of Zhejiang University School of Medicine, Hangzhou, China; 3 Department of Infection Disease, Zhuji People’s Hospital, Zhuji, Zhejiang, China; 4 Department of Thoracic Surgery, Zhuji People’s Hospital, Zhuji, Zhejiang, China

**Keywords:** antioxidant defense, mitochondria, NADPH oxidase, oxidative stress, reactive oxygen species, tendon injury

## Abstract

Tendon injuries are increasingly recognized as conditions driven not only by mechanical overload but also by complex molecular imbalances, particularly involving oxidative stress. Recent evidence highlights the central role of reactive oxygen species (ROS), originating primarily from mitochondrial respiration and NADPH oxidase activation, in regulating cellular responses during tendon injury and repair. Mechanical loading and calcium signaling further influence ROS dynamics, exacerbating oxidative damage or modulating adaptive responses depending on context. Tendon cells counteract oxidative insults through a coordinated antioxidant defense network, including superoxide dismutases, catalase, glutathione peroxidases, and peroxiredoxins. However, in pathological states such as tendinopathy or diabetes, this redox balance is often disrupted, leading to sustained inflammation, extracellular matrix degradation, and impaired healing. This review synthesizes current findings on ROS generation, redox-sensitive signaling pathways, and the functional consequences of oxidative stress in tendon biology. Furthermore, it explores therapeutic strategies targeting redox imbalance, including pharmacological antioxidants and bioengineered scaffolds with antioxidant properties. Understanding these mechanisms provides critical insights into tendon pathophysiology and highlights promising avenues for redox-based regenerative therapies.

## 1 Introduction

**FIGURE 1:**
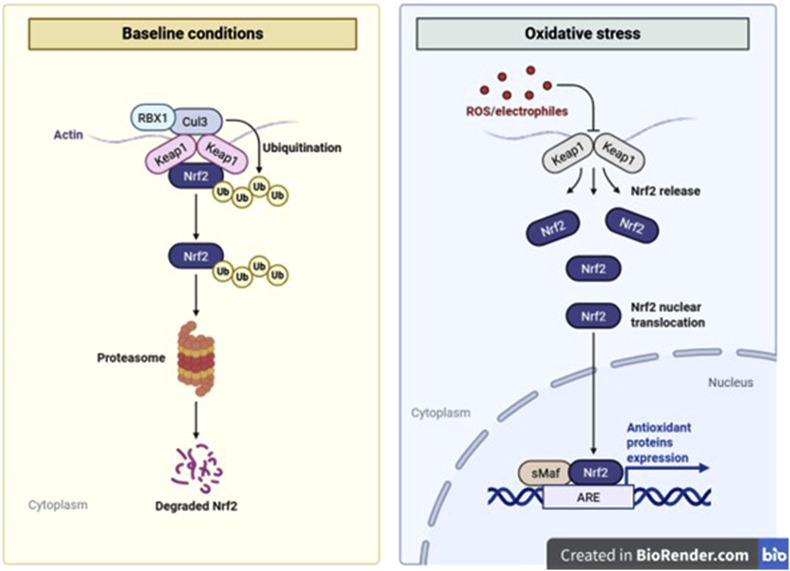
Structural organization of the Achilles tendon and key alterations in pathological tendinopathy.

Tendons are dense, collagen-rich connective tissues that have a critical biomechanical role in transmitting force from muscle to bone, and they are characterized by low cellularity and limited vascularization. In addition, their structure is primarily composed of type I collagen fibrils aligned in a hierarchical organization. This composition provides exceptional tensile strength, but the very features that confer mechanical resilience also render tendons particularly vulnerable to poor regenerative responses following injury (Thorpe and Screen, 2016; Cook et al., 2016). The extracellular matrix (ECM) of healthy tendons is maintained by tenocytes, specialized fibroblastic cells embedded within the matrix, yet this cellular population is sparse, and metabolic exchange is constrained by the tissue’s hypovascular nature (Figure 1) (Siadat et al., 2021; Screen et al., 2015). Mechanical stress is a central aspect of tendon physiology. During normal activity, tendons are subjected to continuous loading, which promotes minor ECM remodeling and microdamage repair. However, excessive or repetitive loading may exceed the intrinsic adaptability of tendon tissues, triggering degeneration and structural compromise (Galloway et al., 2013). The resulting injury spectrum includes both acute ruptures and chronic tendinopathies, often compounded by systemic conditions such as rheumatologic disorders or diabetes (Lipman et al., 2018; Abate et al., 2013). Derwin et al. reported that acute rotator cuff repairs in canine models frequently fail due to high mechanical loading, leading to scar formation rather than true tendon reattachment (Derwin et al., 2007). Healing in such cases is hindered by the tissue’s limited regenerative potential and is typically organized into three overlapping phases; inflammation, proliferation, and remodeling. Each phase involves tightly regulated cellular and molecular responses aimed at restoring function, but these responses are often impaired by persistent oxidative stress (Chartier et al., 2021; Alhaskawi et al., 2025). Reactive oxygen species (ROS) act as key regulators of the molecular pathways that drive tendon degeneration and coordinate tissue repair (Prasetia et al., 2023). Under homeostatic conditions, ROS act as signaling intermediates that influence cell proliferation, differentiation, and ECM turnover. However, pathological elevations in ROS, whether from mechanical overload, ischemia-reperfusion, pharmacologic agents, or chronic inflammation, contribute to oxidative damage (Lennicke and Cochemé, 2021). Elevated ROS levels disrupt cellular organelles, induce stress response pathways, and activate apoptosis and fibrotic signaling, collectively impeding effective tendon healing (Bestwick and Maffulli, 2004).

Understanding the sources and regulation of ROS in tendons, including the roles of nicotinamide adenine dinucleotide phosphate (NADPH) oxidases, mitochondria, and the hypoxia-inducible factor (HIF), is crucial for distinguishing between their beneficial and harmful effects. Furthermore, exploring the crosstalk between calcium signaling and ROS generation under mechanical stress, and the exacerbating effects of conditions such as diabetes, offers insights into the complex molecular landscape of tendon pathology.

This review aims to elucidate the precise role of ROS in tendon injury and repair, with an emphasis on redox homeostasis, molecular signaling pathways, and therapeutic strategies targeting oxidative stress. By dissecting the mechanistic underpinnings and evaluating current antioxidant interventions, we aim to clarify how modulating ROS levels can improve outcomes in tendon healing.

## 2 Sources of ROS in tendon tissue

### 2.1 General principles of cellular ROS generation

ROS are continuously produced in aerobic cells as a natural consequence of oxygen metabolism and regulated enzymatic activity. The principal ROS in biological systems include superoxide anion (O_2_^−^), hydrogen peroxide (H_2_O_2_), and hydroxyl radical (•OH), which differ in reactivity, stability, and diffusion properties (Lennicke and Cochemé, 2021; Rauf et al., 2024). In addition, ROS formation results from the partial reduction of molecular oxygen, a process intrinsic to cellular respiration and various oxidase reactions. Superoxide is typically generated through one-electron oxygen reduction and is rapidly converted into H_2_O_2_ by superoxide dismutases (SODs) (Juan et al., 2021). Furthermore, H_2_O_2_, owing to its relative stability and membrane permeability, acts as a central redox intermediate that modulates intracellular signaling pathways at controlled physiological levels; however, at elevated concentrations, it can exert cytotoxic effects by promoting oxidative damage to proteins, lipids, and nucleic acids (Sies, 2017; Sies and Jones, 2020). In contrast, •OH is highly reactive and primarily associated with oxidative damage rather than regulated signaling. Multiple intracellular systems contribute to ROS generation, including the mitochondrial electron transport chain (ETC.), NADPH oxidases, and other oxidoreductase enzymes. The relative contribution of each source depends on cellular context, metabolic activity, and external stimuli (Zhao et al., 2019; Palma et al., 2024). Importantly, ROS are not inherently pathological. At controlled levels, they function as signaling molecules that regulate kinase activity, transcription factor activation, and adaptive cellular responses. Oxidative stress occurs when the production of ROS exceeds the capacity of cellular antioxidant systems to control redox balance and prevent oxidative damage through mechanisms such as ROS scavenging, metal ion chelation, modulation of redox signaling pathways, and activation of endogenous antioxidant defenses (Liu et al., 2020). The biological consequences of ROS accumulation are therefore governed more by concentration, duration, and antioxidant buffering capacity than by the specific source of generation (Lennicke and Cochemé, 2021). These principles are particularly relevant to tendon tissue, where low vascularization, a hypoxia-prone microenvironment, and the dense collagen-rich ECM can influence ROS production and clearance without changing the fundamental mechanisms of ROS generation.

### 2.2 Major intracellular sources of ROS in tendon

**FIGURE 2:**
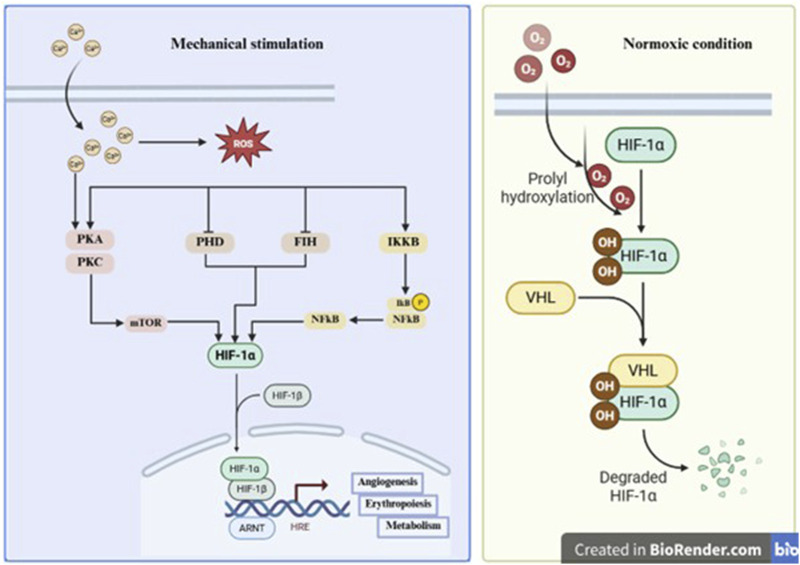
Sources of mitochondrial ROS and the corresponding antioxidant systems.

Mitochondria are the principal organelles responsible for aerobic energy production via oxidative phosphorylation, and play a crucial role in tendon homeostasis due to the high metabolic demand of tenocytes (Casanova et al., 2023). However, mitochondria are also a significant source of ROS, which are produced as byproducts of electron leakage during, ETC., activity. Although several mitochondrial sites can generate ROS, complexes I and III are considered the major contributors (Zhao et al., 2019; Lui et al., 2024). Under physiological conditions, these ROS act as secondary messengers involved in redox signaling, modulating processes such as differentiation, matrix turnover, and cellular adaptation to mechanical load (Sun et al., 2025). Furthermore, during normal mitochondrial respiration, approximately 1%–2% of electrons leak from the, ETC., reducing oxygen prematurely to form O_2_^−^. These are rapidly converted to H_2_O_2_ by manganese superoxide dismutase (MnSOD) in the mitochondrial matrix. While H_2_O_2_ is less reactive than other ROS, it can diffuse through membranes and modulate nuclear transcription, cytoskeletal reorganization, and ECM synthesis in tenocytes (Figure 2) (Lui et al., 2024; Nolfi-Donegan et al., 2020). In tendon cells, mitochondrial ROS generation is influenced by several intrinsic and extrinsic factors. Elevated mitochondrial membrane potential (ΔΨm), a condition associated with energy surplus, ETC., dysregulation, enhances electron leakage and ROS production (Cheng et al., 2024). Mechanical loading, which is intrinsic to tendon physiology, can modulate mitochondrial respiration and bioenergetic demand, thereby influencing, ETC., flux and ROS generation. Additionally, intracellular calcium overload, commonly triggered by mechanical overuse, trauma, or inflammatory stimuli, promotes calcium influx into mitochondria, further disrupting, ETC., function and accelerating ROS formation (Chandra et al., 2021). Furthermore, mitochondrial ROS generation is also modulated by mitochondrial quality control mechanisms, including fission and fusion dynamics, mitophagy, and the activity of uncoupling proteins (Ježek et al., 2018). Mild mitochondrial uncoupling can reduce ΔΨm and limit superoxide production, whereas impaired mitochondrial turnover or structural destabilization may exacerbate ROS leakage. In tendon tissue, which is characterized by low vascularity and fluctuating oxygen availability, mitochondrial function may be particularly sensitive to metabolic perturbations, thereby influencing redox homeostasis (Cheng et al., 2024; Kračun et al., 2025; Wu et al., 2024). Importantly, although mitochondrial ROS originate within the, ETC., the downstream biological effects associated with oxidative signaling are not exclusively determined by their site of production. Given the diffusibility of H_2_O_2_ and the convergence of redox-sensitive pathways, ROS derived from mitochondria may intersect functionally with those generated by NADPH oxidases or other enzymatic systems. Therefore, it is essential to distinguish between mitochondrial ROS as a source of oxidant production and mitochondrial dysfunction as a broader pathological process involving impaired ATP synthesis, altered bioenergetics, and sustained oxidative imbalance. In addition to mitochondria, NADPH oxidases (NOX enzymes) are a major enzymatic source of ROS in non-phagocytic cells, including tenocytes and tendon-derived stem/progenitor cells (Henríquez-Olguín et al., 2019). Unlike mitochondrial ROS, which are produced as metabolic byproducts, NOX enzymes generate ROS in a tightly regulated and deliberate manner as part of redox signaling networks (Magnani and Mattevi, 2019; Pecchillo Cimmino et al., 2023). In tendon tissues, NADPH oxidases, particularly NOX1, NOX2, and NOX4, have emerged as key contributors to both physiological signaling and pathological oxidative stress in response to mechanical loading, inflammation, and metabolic dysregulation (Ferreira and Laitano, 2016; Brandes et al., 2014). Mechanical stretch can activate NOX enzymes through calcium-dependent signaling pathways and protein kinase-mediated phosphorylation of regulatory subunits. Pro-inflammatory cytokines such as interleukin-1β and tumor necrosis factor-α further upregulate NOX expression and enhance enzymatic activity. Hyperglycemic conditions, frequently associated with metabolic disorders affecting tendon integrity, have also been shown to increase NOX1 and NOX4 expression, thereby augmenting intracellular ROS generation (Vermot et al., 2021; Ackerman et al., 2021). Subcellular localization confers additional regulatory specificity to NOX-mediated ROS production. NOX2, typically localized in the plasma membrane, has been shown to mediate oxidative bursts in response to acute injury or immune activation, whereas NOX4, which localizes to intracellular compartments such as the endoplasmic reticulum and mitochondria-associated membranes, is constitutively active and predominantly produces H_2_O_2_. This distinct localization and kinetic profile allows NOX4 to modulate intracellular redox tone and gene expression in a sustained fashion (Pecchillo Cimmino et al., 2023; Begum et al., 2022).

## 3 Biological effects of ROS in tendon tissue

ROS exert multifaceted biological effects in tendon tissue, acting as regulators of intracellular signaling while contributing to degenerative remodeling when redox balance is disrupted. Importantly, the downstream consequences of ROS accumulation are determined primarily by their concentration, duration, spatial distribution, and the efficiency of antioxidant buffering systems rather than exclusively by their site of generation (Ansari et al., 2025). The biological impact of ROS therefore depends on quantitative and temporal parameters. Transient and spatially restricted ROS elevations can participate in regulated redox signaling, whereas sustained increases that exceed antioxidant buffering capacity lead to oxidative stress and structural damage. This distinction reflects the dynamic balance between ROS production and activation of endogenous antioxidant and repair mechanisms (Hong et al., 2024; Chen et al., 2025). ROS act as upstream regulators of degradative enzymes such as matrix metalloproteinases (MMP-1, MMP-3, and MMP-13), which contribute to the breakdown of type I collagen, a key structural component of the tendon ECM. This enzymatic degradation disrupts fibril alignment and mechanical integrity, hallmark features of tendinopathic tissue (Young et al., 2019; Nappi, 2025). Beyond matrix remodeling, mitochondrial ROS directly impact tenocyte viability and phenotype. Accumulation of ROS within the mitochondrial matrix can impair oxidative phosphorylation efficiency, leading to decreased ATP production and opening of the mitochondrial permeability transition pore (mPTP) (Zorov et al., 2014; Chen S. et al., 2024). The subsequent release of pro-apoptotic factors, including cytochrome c, activates caspase-dependent cell death pathways, promoting tenocyte apoptosis in degenerative lesions. Simultaneously, sub-lethal levels of ROS have been shown to induce tenocyte senescence, marked by cell cycle arrest and secretion of pro-inflammatory mediators (Yuan et al., 2003; Worsfold et al., 2024). In addition, mitochondrial ROS act as potent activators of redox-sensitive transcription factors, particularly nuclear factor-κB (NF-κB) and activator protein-1 (AP-1), which amplify inflammatory signaling within the tendon microenvironment (Müller-Eigner and Wojtovich, 2025). This leads to elevated expression of cytokines such as IL-1β, IL-6, and TNF-α, fostering a chronic inflammatory milieu that hinders tissue resolution and favors fibrosis (Palma et al., 2024; Müller-Eigner and Wojtovich, 2025). Notably, the interaction between mitochondrial ROS and mechanical loading exacerbates tissue damage, as ROS amplify mechanotransduction signals that sensitize tendons to overload-induced injury (Gehwolf et al., 2025; Amorim et al., 2022; De Luca et al., 2025). Thus, under conditions of sustained redox imbalance, mitochondrial ROS can act as significant contributors to matrix degradation, inflammation, and tenocyte dysfunction, and fibrotic remodeling in tendon tissue. Their cumulative impact underlies both the initiation and progression of tendinopathy, positioning mitochondrial oxidative stress as a core therapeutic target in tendon-related diseases (De Luca et al., 2025; Gao et al., 2024). Nevertheless, NOX-mediated ROS are involved in the activation of signaling pathways such as ERK1/2, p38 MAPK, and JNK, which influence tenocyte differentiation and mechanotransduction (Jiang et al., 2011; Rastogi et al., 2017). However, in pathological settings, many studies have demonstrated that NOX4 overexpression in tenocytes results in increased expression of matrix MMPs and pro-inflammatory cytokines, including IL-6 and MCP-1, both of which are implicated in tendon fibrosis and degeneration (Mukohara et al., 2021). *In vivo*, inhibition of NOX activity using pharmacological agents such as apocynin or VAS2870 has been shown to attenuate tissue inflammation, reduce collagen fragmentation, and improve histological repair in animal models (Reis et al., 2020; Boshtam et al., 2021). For example, Li et al. reported that scaffold-based delivery of NOX4 inhibitors significantly reduced oxidative stress markers and improved tendon tensile strength following rotator cuff injury in rats (Li et al., 2025a). Moreover, combined approaches using NOX inhibition and antioxidant biomaterials have demonstrated synergistic effects in restoring ECM organization and cellular redox balance (Li et al., 2025a).

## 4 Mechanisms of antioxidant defense and ROS neutralization in tendon

**FIGURE 3:**
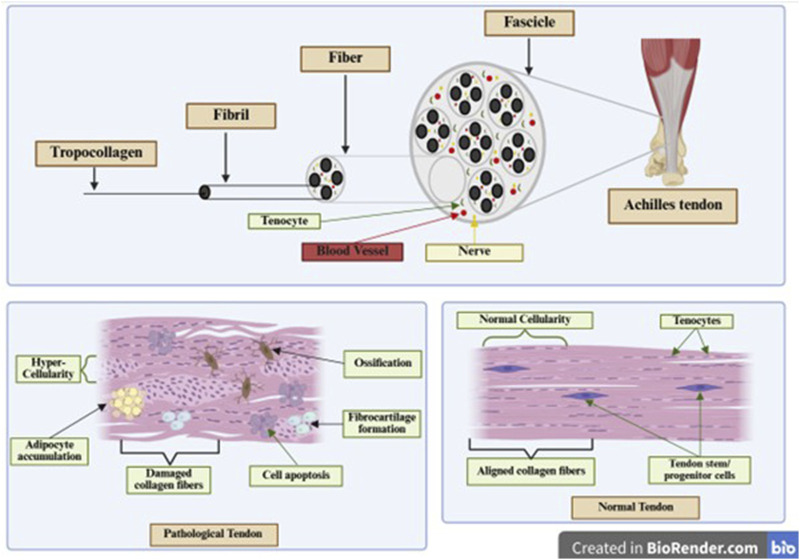
Role of superoxide dismutase (SOD) isoforms in regulating ROS flux and redox signaling.

Tendon cells employ both enzymatic and non-enzymatic antioxidant systems that help regulate cellular redox balance and limit oxidative damage. Among these, enzymatic antioxidants represent the first and most immediate line of defense against excessive ROS generated during mechanical loading, inflammation, hypoxia-reoxygenation, and metabolic stress (Irato and Santovito, 2021; Kozlov et al., 2024). Key enzymatic components include SODs, catalase (CAT), glutathione peroxidases (GPx), and peroxiredoxins (PRDXs) (Ighodaro and Akinloye, 2018). SODs initiate ROS detoxification by catalyzing the conversion of O_2_^−^, primarily generated by mitochondrial electron transport or NADPH oxidase activity, into H_2_O_2_ (Wang et al., 2018). In addition, SOD exists in three isoforms: SOD1 (cytoplasmic), SOD2 (mitochondrial, also known as MnSOD), and SOD3 (extracellular) (Figure 3) (Fukai and Ushio-Fukai, 2011).

**FIGURE 4:**
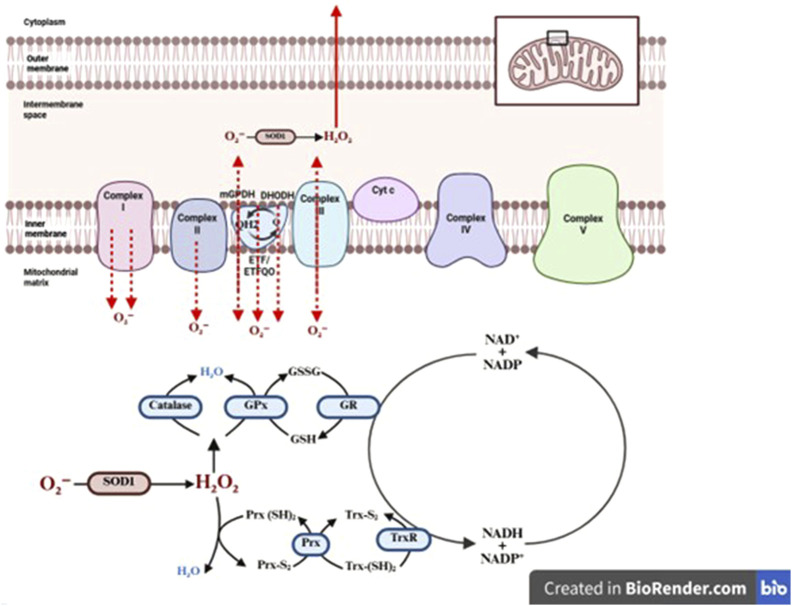
NRF2-Keap1 redox regulation and induction of antioxidant defences.

Among them, SOD2 has a critical role in tendon homeostasis due to its localization within mitochondria, where it mitigates oxidative stress generated during electron leakage. This function is particularly important under mechanical strain or ischemic conditions, which may arise in tendons due to their relatively low vascularity and transient reductions in blood flow during repetitive loading, leading to hypoxia and increased mitochondrial ROS production (Ostrom et al., 2025; Flynn and Melov, 2013). Reduced expression or activity of SOD2 has been observed in tendinopathic tissues and diabetic tendons, correlating with increased oxidative damage, mitochondrial dysfunction, and tenocyte apoptosis (Jiang et al., 2016). Itoigawa et al. investigates the role of oxidative stress, specifically SOD, in the recurrence of rotator cuff tears after surgical repair. The study demonstrates that elevated levels of SOD, as measured by dihydroethidium fluorescence and SOD activity, are significantly associated with increased risk of tendon retear (Itoigawa et al., 2021). A study by Uehara et al. showed that aging significantly reduces the expression of mitochondrial SOD2 in rat Achilles tendons. The aged tendon group showed markedly lower SOD2 mRNA levels and diminished SOD2-positive staining in tenocytes, which coincided with increased oxidative damage, mitochondrial swelling, and disruption of cristae structure under transmission electron microscopy (Uehara et al., 2022). Furthermore, H_2_O_2_ generated from SOD activity is further degraded by CAT, which decomposes it into water and molecular oxygen. CAT is predominantly localized in peroxisomes and operates efficiently under conditions of high H_2_O_2_ flux (Rasheed, 2024). In addition, CAT role becomes particularly important during acute oxidative stress, where rapid H_2_O_2_ clearance is necessary to prevent its conversion into highly reactive OH via Fenton chemistry (Abdalbagemohammedabdalsadeg et al., 2024; Gebicka and Krych-Madej, 2019). Experimental studies in animal models of tendon overuse and inflammation have shown that CAT expression is upregulated during early healing phases but declines under chronic oxidative conditions, contributing to sustained ROS accumulation (Radák et al., 2002). In a study investigating oxidative stress regulation in tendon-derived cells, Yin et al. highlighted the critical role of CAT in maintaining redox balance within tendon tissue. The findings revealed a significant downregulation of CAT expression in degenerative tendons, particularly under conditions of elevated inflammation and oxidative stress (Yin et al., 2025). Gallorini et al. found that inflammatory cytokines significantly suppress CAT expression in human tendon stem/progenitor cells (TSPCs), leading to elevated H_2_O_2_ levels and oxidative stress. This impaired catalase activity compromised the cells’ antioxidant defense, promoting redox imbalance, cellular senescence, and altered tendon matrix remodeling (Gallorini et al., 2020). Another vital enzymatic system includes the GPx, which catalyze the reduction of both H_2_O_2_ and lipid hydroperoxides to their corresponding alcohols, using reduced GSH as a cofactor (Pei et al., 2023; Shen Y. et al., 2025). Liang et al. showed that quercetin treatment significantly increased GPx activity in injured rat tendons, which was associated with reduced oxidative damage and tendon adhesion. While GSH levels were not directly measured, the enhanced GPx response implies activation of the GSH-dependent antioxidant system, highlighting its protective role in tendon healing (Liang et al., 2020). Another study presented that activation of SIRT3 significantly increased GPx expression and activity in injured Achilles tendons. This upregulation reduced oxidative damage by lowering ROS and lipid peroxidation levels, while preserving mitochondrial integrity (Furuta et al., 2023). Nevertheless, PRDXs form an additional tier of antioxidant defense, especially in regulating redox signaling under physiological ROS levels. These thiol-specific peroxidases reduce H_2_O_2_, peroxynitrite (ONOO^−^), and alkyl hydroperoxides via conserved cysteine residues, often acting in tandem with the thioredoxin (Trx) system (Neumann et al., 2009; Elko et al., 2019). Guo et al. identifies a pathological tendon-derived stem cell subset defined by low PRDX2, elevated inflammation, impaired proliferation and migration, and increased senescence. Loss of PRDX2 drives TNF pathway activation and excessive ROS, worsening microenvironmental deterioration and disease progression (Guo et al., 2023). Yuan et al. demonstrates that although endogenous PRDX5 increases after H_2_O_2_ exposure, this natural response is insufficient to prevent oxidative damage. However, forced overexpression of PRDX5 significantly protects tendon cells, reducing H_2_O_2_-induced apoptosis by nearly half and preventing the decline in collagen synthesis (Yuan et al., 2004). Meng et al. found that catechol-based PEG-D4 hydrogels generate substantial H_2_O_2_ during oxidative crosslinking, which increases network stiffness, causes localized cytotoxicity, elevates PRX2 expression in dermal and tendon fibroblasts, and *in vivo* promotes superoxide production, macrophage recruitment, and M2 polarization, identifying H_2_O_2_ as a central mediator of both material properties and biological responses in mussel-inspired biomaterials (Meng et al., 2017). The coordinated activity of these enzymatic antioxidants is essential for preserving the structural integrity and cellular viability of tendon tissues. Dysregulation of these systems, whether through genetic, inflammatory, or metabolic perturbations disrupts redox equilibrium and initiates a cascade of oxidative injury, tenocyte dysfunction, and ECM degradation, all of which underpin the progression of tendinopathy. In contrast, tendon cells also rely on a diverse repertoire of non-enzymatic antioxidants that function as both direct radical scavengers and essential cofactors for enzymatic detoxification pathways (Mirończuk-Chodakowska et al., 2018). Trx-thioredoxin reductase (TrxR) axis works synergistically with PRDX to detoxify peroxides and maintain thiol homeostasis in proteins (Garcia et al., 2012). Trx reduces disulfide bonds in oxidized proteins, thereby reversing oxidative modifications that can impair enzymatic function and signaling. This system also plays a role in regulating the activity of redox-sensitive transcription factors, such as NF-κB and AP-1, which mediate inflammatory responses in tendon injury (Muri and Kopf, 2023; Hasan et al., 2022). Studies have shown that modulation of the Trx system in tenocytes under high-glucose or inflammatory conditions can reduce cytokine release and restore ECM gene expression, underscoring its relevance in tendon redox balance (Tsaklis et al., 2015). In addition to endogenous molecules, micronutrient-derived antioxidants, particularly vitamin C (ascorbic acid) and vitamin E (α-tocopherol), have critical roles in tendon antioxidant defense (Noriega-González et al., 2022; Rezaei, 2010). Vitamin C acts as a direct neutralizer of ROS and also serves as a cofactor for prolyl and lysyl hydroxylase enzymes, which are essential for the post-translational hydroxylation of proline and lysine residues in collagen, thus stabilizing the triple helix structure of type I collagen, the principal load-bearing component of tendon (Kietzmann, 2023; Alberts et al., 2025). In addition, vitamin E, a lipid-soluble antioxidant, is embedded in cellular membranes where it protects polyunsaturated fatty acids from lipid peroxidation (Traber et al., 1995; Rizvi et al., 2014). Both vitamins have been shown to exert cytoprotective effects in oxidative stress models of tendon injury, and their supplementation has been associated with enhanced collagen synthesis and improved biomechanical outcomes in tendon repair studies (Tack et al., 2018; Plasencia et al., 1999). Collectively, these non-enzymatic antioxidant systems work in concert with enzymatic defenses to maintain redox homeostasis, regulate redox-sensitive signaling pathways, and support cellular and matrix integrity in tendons. Dysregulation or depletion of these molecules compromises the cell’s ability to neutralize ROS, contributing to oxidative damage, impaired healing, and progression of chronic tendinopathies (Irato and Santovito, 2021; Sachdev et al., 2023). Therefore, enhancing the availability or function of these non-enzymatic antioxidants may offer a promising strategy for redox-targeted interventions in tendon repair. The activity and expression of antioxidant systems in tendon tissue are tightly regulated by redox-sensitive transcription factors that enable cells to dynamically respond to oxidative stress. The Nuclear factor erythroid 2-related factor 2 (NRF2) a main regulator of the cellular antioxidant response (Ngo and Duennwald, 2022). Under basal conditions, NRF2 is constitutively suppressed through its interaction with Kelch-like ECH-associated protein 1 (KEAP1), which serves as a substrate adaptor for the CUL3–RBX1 E3 ubiquitin ligase complex and promotes continuous ubiquitination and proteasomal degradation of NRF2 (Bryan et al., 2013). Foundational studies demonstrated that KEAP1 binds the Neh2 domain of NRF2 and maintains low steady-state NRF2 activity in unstressed cells, thereby preventing unnecessary activation of cytoprotective genes (Itoh et al., 1999; Kobayashi et al., 2006). Upon oxidative or electrophilic stress, specific cysteine residues within KEAP1 undergo covalent modification, leading to conformational changes that impair KEAP1-mediated ubiquitination of NRF2. As a result, newly synthesized NRF2 accumulates in the cytoplasm, translocates to the nucleus, heterodimerizes with small Maf proteins, and binds to antioxidant response elements (AREs) in the promoter regions of target genes (Ishii et al., 2022; Nguyen et al., 2009). This canonical activation mechanism enables NRF2 to function as a redox-sensitive transcriptional switch that dynamically adjusts cellular antioxidant capacity according to oxidative burden. The antioxidant effects of NRF2 are mediated not by direct ROS scavenging, but by transcriptional upregulation of an integrated network of cytoprotective genes that enhance ROS neutralization and redox homeostasis. A central component of this program is the glutathione (GSH) system. NRF2 induces the catalytic and modifier subunits of glutamate-cysteine ligase (GCLC and GCLM), the rate-limiting enzyme in GSH biosynthesis, as well as the cystine/glutamate antiporter (SLC7A11), which increases intracellular cysteine availability (He et al., 2020; Vomund et al., 2017). In addition, NRF2 regulates glutathione reductase (GSR), thereby maintaining the reduced GSH pool required for detoxification of hydrogen peroxide and lipid peroxides. Experimental evidence demonstrates that disruption of NRF2 signaling markedly compromises cellular GSH redox balance and increases susceptibility to oxidative injury (Harvey et al., 2009). Beyond the GSH axis, NRF2 also enhances the Trx system by regulating thioredoxin and TrxR expression, contributing to the reduction of oxidized protein thiols and peroxides. Furthermore, NRF2 induces NAD(P)H: quinone oxidoreductase 1 (NQO1), multiple glutathione S-transferases (GSTs), and heme oxygenase-1 (HMOX1), collectively limiting electrophile accumulation, reducing redox cycling, and mitigating oxidative damage (Figure 4) (Jaganjac et al., 2020; Lu et al., 2016). In tissues subjected to mechanical stress and inflammatory stimuli, NRF2 functions as an adaptive defense mechanism that determines whether ROS act as signaling mediators or progress toward pathological oxidative stress. By increasing the buffering capacity of tenocytes, NRF2 activation preserves mitochondrial integrity, limits lipid peroxidation, and reduces activation of ROS-amplifying cell death pathways, including apoptosis and ferroptosis (Kasai et al., 2020). Conversely, NRF2 deficiency exacerbates ROS accumulation and induces senescence and apoptosis in tenocytes, especially in diabetic and aged tendons (Chen D. et al., 2024; Fragoulis et al., 2023). In parallel, Forkhead box O (FOXO) transcription factors, particularly FOXO3a, play an important role in antioxidant regulation. FOXO proteins are activated in response to oxidative stress, nutrient deprivation, or growth factor withdrawal, and promote the transcription of antioxidant enzymes (Rodriguez-Colman et al., 2024; Webb and Brunet, 2014). Dysregulation of FOXO signaling has been linked to increased oxidative damage, reduced stemness, and impaired matrix remodeling (Bernardo et al., 2023). FOXO proteins also intersect with metabolic sensors such as AMP-activated protein kinase (AMPK), which becomes activated under energy-depleted or hypoxic conditions commonly found in avascular tendon zones (Kodani and Nakae, 2020; Greer et al., 2007). Furthermore, signaling pathways such as PI3K/Akt and MAPK modulate the nuclear translocation and transcriptional activity of NRF2 and FOXO (Hammad et al., 2023). Generally, the dynamic regulation of antioxidant defenses in tendon involves a coordinated network of transcription factors and kinases that integrate oxidative cues, mechanical signals, and metabolic stress. Enhancing these regulatory pathways, either pharmacologically (e.g., NRF2 activators such as sulforaphane or bardoxolone) or via biomechanical conditioning, may offer novel therapeutic strategies to strengthen tendon resilience against oxidative stress and improve healing outcomes (Lu et al., 2023; Houghton et al., 2016).

## 5 Hypoxia-inducible factor-mediated cellular responses in tendon

**FIGURE 5:**
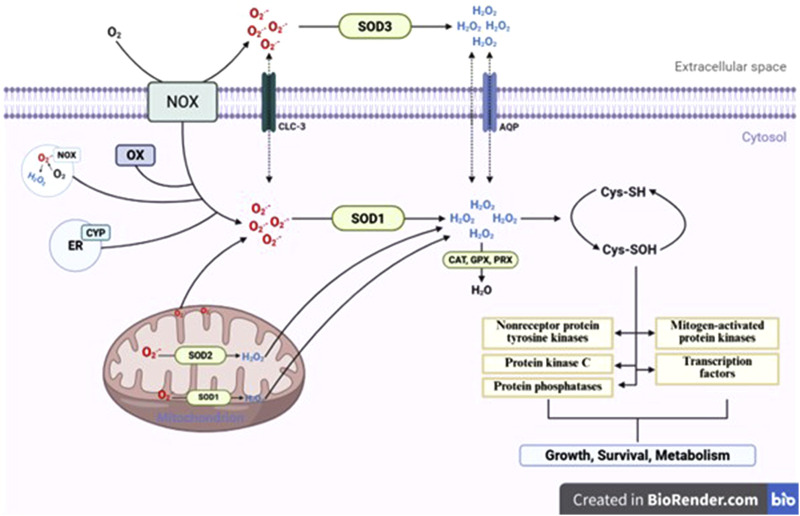
Mechanically induced stabilization of HIF-1α and its degradation under normoxia.

The HIF signaling pathway serves as a key regulator of cellular adaptation to hypoxia. The HIF family comprises transcription factors that act as regulators of oxygen homeostasis (Kumar and Choi, 2015). HIF-1α is the most well-characterized and has been implicated in a wide range of biological processes relevant to tendon physiology and pathology, including angiogenesis, metabolism, oxidative stress regulation, ECM remodeling, and inflammation (Bakleh and Al Haj Zen, 2025; Shahid et al., 2024). Under normoxic conditions, HIF-1α is rapidly hydroxylated by prolyl hydroxylase domain (PHD) enzymes, which promotes its recognition by the von Hippel–Lindau (VHL) E3 ubiquitin ligase complex, leading to ubiquitination and subsequent proteasomal degradation (Zhang et al., 2025). However, during hypoxia, a common microenvironment in both acute tendon injury and chronic tendinopathy, PHD activity is suppressed due to limited oxygen availability, allowing HIF-1α to escape degradation (Wang et al., 2022). Stabilized HIF-1α translocates to the nucleus, dimerizes with HIF-1β (ARNT), and binds to hypoxia-responsive elements in the promoters of target genes (Figure 5). This transcriptional program activates a broad array of genes involved in angiogenesis (e.g., VEGF, ANGPT1), glycolytic metabolism (e.g., GLUT1, PDK1), survival (e.g., BNIP3), and matrix remodeling (e.g., MMPs, TIMPs) (Zhang et al., 2025; Menon et al., 2018; Yu D. et al., 2016).

In the context of tendon biology, HIF-1α expression is markedly elevated during the early phase of tendon injury, where it promotes angiogenic signaling and metabolic reprogramming to support repair in hypoxic zones (Millar et al., 2012). Notably, VEGF, a canonical HIF-1α target, enhances neovascularization at injury sites, facilitating the influx of inflammatory and progenitor cells (Bakleh and Al Haj Zen, 2025; Ramakrishnan et al., 2014; Peter et al., 2004; Zulkifli et al., 2023). Recent studies have highlighted a role for HIF-1α in tenocyte differentiation and stem cell fate. Yu et al. showed that hypoxia markedly enhances the tenogenic differentiation of adipose-derived mesenchymal stem cells in co-culture with tenocytes by upregulating HIF-1α, while HIF-1α inhibition suppresses this response, and FG-4592 mediated stabilization of HIF-1α further amplifies it, identifying HIF-1α as a key driver of adipose-derived mesenchymal stem cell commitment toward a tenocyte lineage (Yu Y. et al., 2016). However, prolonged hypoxia or aberrant HIF-1α signaling can shift differentiation toward non-tenogenic lineages, including adipogenic or chondrogenic fates, thereby impairing functional regeneration. From a therapeutic perspective, modulating the HIF pathway presents a double-edged sword. While controlled activation of HIF-1α (e.g., via PHD inhibitors like DMOG) can enhance early healing and angiogenesis, chronic or unregulated activation promotes fibrosis, ECM disorganization, and persistent inflammation (Wu et al., 2018; Chen M.-h. et al., 2021). Therefore, spatiotemporal regulation of HIF activity is essential in tendon healing strategies, and biomaterials capable of delivering hypoxia-responsive cues or transiently stabilizing HIF-1α may offer targeted therapeutic advantages.

## 6 Tendon injury

### 6.1 Mechanosensitive calcium pathways and ROS generation in tendon injury

Calcium ions (Ca^2+^) are regulators of tendon cell signaling, acting as key second messengers that mediate processes such as cytoskeletal dynamics, ECM remodeling, oxidative balance, and apoptotic signaling (Bootman and Bultynck, 2020). Intracellularly, calcium is stored and regulated within the ER and mitochondria. The ER releases calcium *via* IP_3_ and ryanodine receptors, while reuptake is governed by SERCA pumps. Mitochondria buffer excess cytosolic calcium through the mitochondrial calcium uniporter (MCU), coupling it to ATP production. However, calcium overload in mitochondria impairs, ETC., function, elevates ROS levels, and triggers mPTP opening, initiating intrinsic apoptotic cascades (Zhao et al., 2019; Yu Y. et al., 2016). Calcium homeostasis is maintained through a complex interplay between plasma membrane calcium channels, intracellular calcium stores, and calcium-binding proteins (Bagur and Hajnóczky, 2017). Mechanically induced calcium influx primarily occurs through stretch-activated channels, including members of the transient receptor potential (TRP) family such as TRPV4, TRPC1, and TRPM7, as well as Piezo1, a mechanosensitive channel implicated in tendon mechanotransduction (Ji et al., 2002; Vangeel and Voets, 2019; Zhang et al., 2023). These channels open in response to membrane deformation or shear stress, allowing extracellular calcium to enter the cytosol and activate downstream pathways such as calmodulin-CaMKII, MAPK, and calcineurin-NFAT signaling (Park et al., 2020; Raffaello et al., 2016). In the physiological conditions, this promotes tenogenic gene expression and matrix maintenance. However, under excessive or repetitive strain, calcium overload through TRPV4 and Piezo1 triggers activation of NADPH oxidase (NOX) enzymes and mitochondrial ROS production, leading to oxidative damage, inflammatory gene expression, and cell apoptosis (Swain and Liddle, 2021). In addition, the calcium influx activates protein kinase C (PKC) and calmodulin, which are essential for the assembly and activation of the NOX2 complex (Moussa et al., 2025). PKC-mediated phosphorylation of p47phox, a cytosolic NOX subunit, facilitates its translocation to the membrane, where it associates with gp91phox (NOX2) and p22phox, forming the catalytically active oxidase. Simultaneously, calmodulin, when bound to Ca^2+^, modulates the activity of both cytosolic subunits and intermediary kinases, amplifying the NOX2 response (Fontayne et al., 2002; Yang and Tsai, 2022; Feske et al., 2007). Experimental models of cyclic mechanical stretch have demonstrated that inhibition of calcium influx *via* TRPV4 antagonists or calcium chelation markedly suppresses NOX-derived ROS and prevents downstream inflammation and tenocyte apoptosis (Bian et al., 2024). In parallel, voltage-gated calcium channels (VGCCs), such as Cav1.2, contribute to calcium dynamics in response to depolarizing stimuli, particularly in inflamed or aged tendons (Magra et al., 2007).

### 6.2 Diabetic tendon injury and repair

Diabetes mellitus (DM), especially type 2 diabetes, significantly increases the risk of tendon degeneration, impaired healing, and rupture. Tendons from diabetic individuals present with a distinct pathophysiological profile shaped by chronic metabolic dysregulation, low-grade inflammation, and oxidative stress. Among the multiple molecular mechanisms implicated, ROS which has a role in both the initiation and progression of diabetic tendon injury and repair failure (Xu J. et al., 2024). Diabetic tendons exhibit profound structural remodeling, including disorganized collagen alignment, reduced fibril diameter, and a shift from type I to type III collagen (Nichols et al., 2020). Non-enzymatic glycation of matrix proteins leads to the accumulation of advanced glycation end-products (AGEs), which form abnormal cross-links that stiffen the ECM and reduce tendon elasticity (Singh et al., 2014; Pal and Bhadada, 2023). These AGEs activate the receptor for advanced glycation end-products (RAGE) on tendon cells, initiating a redox-sensitive signaling cascade that promotes chronic inflammation and ECM degradation. This process is intimately linked to ROS generation, as AGEs-RAGE interaction induces NADPH oxidase (NOX1, NOX4) expression, triggering sustained superoxide and H_2_O_2_ production that further modifies ECM integrity (Yang et al., 2024; Wautier et al., 2001; Shi et al., 2021). At the cellular level, tenocytes and tendon-derived stem/progenitor cells exposed to hyperglycemia exhibit decreased proliferation, impaired migration, and a blunted capacity for tenogenic differentiation (Vaidya et al., 2023). Chronic exposure to elevated glucose alters transcriptional regulation, suppressing tendon-specific genes such as scleraxis (Scx) and Mohawk (Mkx) while promoting adipogenic and fibrotic markers (Wu et al., 2017; Liu et al., 2015). Furthermore, ROS impairs stem cell fate decisions in tendon-derived stem/progenitor cells, shifting differentiation away from regenerative tenogenesis and toward non-functional fibrotic or adipogenic lineages. This process is exacerbated by hyperglycemia-induced suppression of antioxidant defenses, including GPx and SOD2, leaving diabetic tendon cells more vulnerable to redox imbalance (Wal et al., 2019; Ryu et al., 2015). The repair of injured tendons in diabetic individuals is characterized by delayed granulation tissue formation, reduced cellularity, and disorganized matrix deposition. This impaired healing response is heavily influenced by ROS (Nichols et al., 2019; Shi et al., 2015). Persistent oxidative stress suppresses the angiogenic response, *via* downregulation of VEGF, leading to hypoxia and further ROS accumulation. Additionally, redox-sensitive transcription factors such as NF-κB and AP-1 are persistently activated, driving the expression of pro-inflammatory cytokines (e.g., TNF-α, IL-1β) and MMPs that degrade the already-compromised ECM (Caturano et al., 2023). The result is a chronic inflammatory milieu that replaces regenerative healing with fibrosis and functional impairment. Biomechanical assessments consistently show that diabetic tendons possess significantly reduced ultimate tensile strength, stiffness, and energy to failure, making them more prone to re-injury (Connizzo et al., 2014). Animal models of streptozotocin-induced diabetes or high-fat diet exposure reveal prolonged inflammatory phases and incomplete resolution of the repair cascade (Connizzo et al., 2014; Leguina-Ruzzi et al., 2018). Kurosawa et al. Showed that apocynin, a NOX inhibitor, reduced ROS accumulation, NOX1/NOX4 and IL-6 expression, and apoptosis in rat tenocytes under high-glucose conditions, suggesting its potential for treating diabetic tendinopathy (Kurosawa et al., 2020). Similarly, Yamaura et al. found that NMN enhanced SIRT1/6 expression while suppressing NOX1/NOX4, IL-6, ROS, and apoptosis, thus mitigating oxidative stress both *in vitro* and in a collagenase-induced tendinopathy model (Yamaura et al., 2022). In addition, Yoshikawa et al. found that quercetin, a dietary flavonoid, protected diabetic rat tendons by decreasing NOX1/NOX4 expression, reducing ROS and IL-6, and restoring collagen I/III balance (Yoshikawa et al., 2022).

## 7 ROS in tendon healing

ROS function as dynamic signaling intermediates during tendon healing, regulating the transitions between inflammation, proliferation, and matrix remodeling (Table 1). In the early phase of repair, transient ROS bursts from infiltrating neutrophils and macrophages facilitate the clearance of damaged matrix and apoptotic cells, acting as upstream activators of cytokine release and immune resolution. Within this window, low to moderate ROS levels promote the activation of redox-sensitive transcription factors such as NRF2, AP-1, and STAT3, initiating a transcriptional program that supports angiogenesis, fibroblast migration, and ECM turnover (Soliman and Barreda, 2023; Darrieutort-Laffite et al., 2024). During the proliferative phase, ROS generated predominantly by mitochondrial respiration and NOX family enzymes serve as critical regulators of tenocyte proliferation and TSPC fate (Chartier et al., 2021; Schulze-Tanzil et al., 2022). At physiological levels, ROS mediate cytoskeletal remodeling and focal adhesion dynamics through redox modulation of small GTPases and integrin signaling, facilitating cellular migration into the wound bed. Concurrently, they modulate the activity of MMPs and lysyl oxidase (LOX), orchestrating the degradation and realignment of collagen fibers for neotendon formation (Nakao et al., 2023; Citro et al., 2023). However, the transition to the remodeling phase requires tight attenuation of ROS activity. ROS influence tendon ECM dynamics largely by modulating cytokine-mediated signaling cascades that govern collagen metabolism. Several cytokines exert distinct and sometimes opposing effects on matrix organization (Li et al., 2023; Yang et al., 2021). For example, TNF-α suppresses the accumulation of type I collagen within the matrix, while IL-6 enhances overall collagen synthesis (Ackerman et al., 2021; Wang and Li, 2023). IL-33 and IL-17 adjust the balance of collagen isoforms by elevating the proportion of type III relative to type I collagen and simultaneously stimulating the expression of matrix MMPs that promote matrix remodeling (Cayrol and Girard, 2022; Shen J. et al., 2025). Millar et al. demonstrated that microRNA-29a regulates IL-33-driven collagen remodeling in tendons, acting as a key molecular switch that controls inflammation and the pathological shift from type I to type III collagen during early tendinopathy, via an IL-1 receptor-dependent NF-κB/ERK pathway, thereby contributing to tendon repair (Millar et al., 2015). Meanwhile, the absence of IL-4 disrupts fibrillar alignment and weakens collagen crosslinking, reflecting its stabilizing role in ECM integrity (Andersen et al., 1985; Lin et al., 2005). In addition, persistent oxidative signaling delays matrix maturation, and reduces the mechanical strength of the healing tendon. Mitochondrial quality control mechanisms, including mitophagy and fission/fusion dynamics, are essential at this stage to prevent chronic ROS leakage and restore redox homeostasis. Failure of these processes, as observed in impaired healing models, leads to mitochondrial dysfunction, accumulation of lipid peroxidation products (e.g., 4-HNE), and defective collagen cross-linking (Cheng et al., 2024; Ye et al., 2020). Furthermore, localized bursts of ROS at the tendon-bone interface (enthesis) are necessary for fibrocartilage regeneration and mineralized ECM deposition, suggesting a zonal redox requirement. Additionally, biomechanical loading modulates ROS generation *via* integrin mechanotransduction and calcium influx, which in turn influences ROS-dependent gene expression through mechano-sensitive pathways like YAP/TAZ and MAPK/ERK (Xu Z. et al., 2024; Lei et al., 2021).

**TABLE 1:** Cellular and molecular events across the stages of tendon healing (Alhaskawi et al., 2024; Leong et al., 2020).

Stage of healing	Time course	Principal cellular and matrix events	Key molecular mediators
Inflammatory stage	Hours- days	• Rapid recruitment of inflammatory cells (platelets, neutrophils, monocytes) • Presence of erythrocytes and circulating mesenchymal stem cells • Early matrix disruption and initiation of provisional scaffold formation	• Pro-inflammatory cytokines: IL-6, IL-1β • Growth factors initiating repair: bFGF, IGF-1, PDGF • Matrix-modulating factors: TGF-β • Angiogenic mediator: VEGF
Proliferative stage	Days- weeks	• Increased cellularity and fibroblast proliferation • Deposition of collagen type III as an early repair matrix • Activation of resident tendon stem/progenitor cells (TSPCs)	• Matrix synthesis drivers: bFGF, IGF-1, TGF-β • Tenogenic growth factors: GDF-5, GDF-6, GDF-7 • Angiogenic/mitogenic mediator: VEGF • Cell proliferation factor: PDGF
Remodeling stage	Weeks- Years	• Reduction in cellularity as tissue matures • Transition from collagen type III to collagen type I, restoring tensile strength • Matrix reorganization and alignment	• Tenogenic regulators: GDF-5, GDF-6, GDF-7 • Remodeling and maturation factor: TGF-β • Matrix-stabilizing mediator: IGF-1

## 8 Therapy of tendon injury

### 8.1 Antioxidants in tendon injury and repair

Pharmacological antioxidant therapy has emerged as a promising approach for modulating oxidative stress in tendon injuries, aiming to counteract the deleterious effects of excessive ROS on tendon cells and ECM integrity. Among the most studied agents, N-acetylcysteine (NAC) functions both as a direct ROS scavenger and a precursor for glutathione synthesis, effectively restoring redox balance. Kim et al. found that NAC significantly reduces glutamate-induced oxidative cytotoxicity in rat supraspinatus fibroblasts, preventing apoptosis and calcium influx (Kim et al., 2019). NAC has been shown to protect tenocytes from apoptosis, reduce MMP activity, and enhance type I collagen production under oxidative stress conditions. Lu et al. investigated NAC in TSPCs and a rat Achilles tendon injury model. *In vitro*, 500 µM NAC reduced ROS, enhanced cell proliferation, and upregulated tenogenic markers (SCX, TNC, TNMD, COL1A1) (Lu et al., 2023). Transcriptomic and inhibition experiments demonstrated activation of integrin α5β1–PI3K/AKT signaling. *In vivo*, local NAC administration decreased oxidative stress and improved collagen organization and tendon repair, supporting its therapeutic potential in tendon regeneration. (Lu et al., 2023). Furthermore, Büyükdoğan et al.evaluated systemic NAC (150 mg/kg/day intraperitoneally) in a rat Achilles tenotomy model, focusing on early healing phases (1 and 3 weeks). No significant differences were observed at week 1; however, by week 3, NAC significantly reduced Movin and Bonar histological scores, increased collagen type I expression and collagen I/III ratio, and improved tensile strength and toughness. These findings suggest that NAC primarily enhances early remodeling rather than the initial inflammatory phase (Büyükdoğan et al., 2025). In addition, Aydın et al. evaluated adipose-derived stem cells (ADSCs), systemic N-acetylcysteine (15 mg/kg/day), and their combination in a rabbit chronic rotator cuff tear model after a 6-week degeneration period and surgical repair. At 12 weeks, treated groups showed improved collagen organization, reduced fatty infiltration, and increased type I collagen expression, with the ADSC + NAC group demonstrating the greatest effects. Biomechanical testing confirmed higher tensile strength, particularly with combination therapy, indicating synergistic enhancement of tendon-to-bone healing (Aydın et al., 2025). Along with that, in an ovine collagenase-induced tendinopathy model, local injection of bone marrow-derived mesenchymal stem cells significantly enhanced tendon repair, as evidenced by increased collagen type I deposition and recruitment of CD34^+^ progenitor cells, indicating improved extracellular matrix remodeling and modulation of inflammatory and redox microenvironments during tendon healing (Lacitignola et al., 2014). In a separate ovine rotator cuff repair model, credille et al. evaluated a biphasic acellular interpositional allograft in an ovine rotator cuff repair model and reported progressive collagen organization and partial restoration of the fibrocartilaginous enthesis without excessive inflammatory responses (Credille et al., 2023).

Additionally, vitamin C plays a dual role as a cofactor in collagen synthesis and as a potent antioxidant, promoting ECM stability and attenuating ROS-induced activation of pro-apoptotic signaling pathways (Ueda et al., 2024). Uehara et al. showed that oral N-acetylcysteine and vitamin C reduced oxidative stress, improved histological tendon-to-bone healing, and modulated antioxidant enzymes (PRDX5 and SOD1, respectively) in a rat rotator cuff model [189]. Mienaltowski and colleagues examined vitamin C supplementation in three-dimensional constructs seeded with progenitor cells from the tendon proper and peritenon of the equine superficial digital flexor tendon. Vitamin C reduced pro-inflammatory and degenerative gene expression, including C-X-C motif chemokine ligand 8, bone morphogenetic protein 4, and matrix metalloproteinase 12, while increasing anti-inflammatory tumor necrosis factor alpha-induced protein 3 and extracellular matrix-related genes such as SPARC-related modular calcium-binding protein (Mienaltowski et al., 2023). Moreover, vitamin E, a lipid-soluble antioxidant, protects cell membranes from lipid peroxidation and reduces the expression of inflammatory mediators such as COX-2 and iNOS in tendinopathic tissues. While these general antioxidants have demonstrated efficacy *in vitro* and in animal models, their clinical use is often limited by short half-life and low tissue specificity (Lee et al., 2017; Mişcă et al., 2025). Mitochondria-targeted antioxidants such as MitoQ and SS-31 (elamipretide) have been developed to more precisely modulate intracellular ROS production (Jiang et al., 2020). MitoQ, a coenzyme Q10 analog, accumulates selectively in mitochondria where it neutralizes superoxide generated during electron transport, thereby preserving mitochondrial function and preventing tenocyte apoptosis (Sun et al., 2017; Lowes et al., 2009; Zhang X. et al., 2021). SS-31, a small peptide that binds to cardiolipin on the inner mitochondrial membrane, stabilizes the, ETC., reduces ROS leakage, and improves ATP synthesis. In an *in vitro* study using primary human tenocytes from healthy hamstring and degenerative supraspinatus tendons, degenerative cells exhibited mitochondrial depolarization, reduced mitochondrial number, decreased SOD activity, increased MMP1 and FABP4 expression, and impaired viability. Treatment with 1 μM SS-31 for 72 h restored mitochondrial membrane potential and morphology, normalized SOD activity, reduced catabolic gene expression, and partially improved cell viability, supporting a mitoprotective strategy in tendinopathy (Zhang et al., 2022a). Furthermore, Zhang et al. found that in a murine supraspinatus tendinopathy model, SS-31 (elamipretide) restored mitochondrial structure and function by improving cristae morphology, ATP synthase and SOD2 activity, and collagen organization, thereby enhancing tendon strength and healing, especially when combined with mechanical decompression to synergistically reverse tendinopathic degeneration (Zhang et al., 2022b). Moreover, inhibition of ROS production at the source has gained attention, particularly through NOX (NADPH oxidase) inhibition. GKT137831 (setanaxib), a selective NOX1/NOX4 inhibitor, has demonstrated potent anti-fibrotic and anti-inflammatory effects by reducing oxidative stress-induced MMP expression and TGF-β1 signaling (Oh et al., 2024; Bokhari and Murrell, 2012). In addition to synthetic antioxidants, naturally occurring polyphenols such as curcumin, resveratrol, and quercetin have shown multi-targeted antioxidant effects. Curcumin downregulates NF-κB and MAPK signaling, promoting tenocyte survival and collagen synthesis, while resveratrol activates SIRT1-mediated mitochondrial protection and supports angiogenesis in oxidative microenvironments (Zhang et al., 2024). Both compounds enhance endogenous antioxidant responses via NRF2 activation and have been integrated into nanoformulations to improve their bioavailability and therapeutic index. Chen et al. found that in a rat rotator cuff tear model, a self-healing hydrogel releasing curcumin and Mg^2+^ synergistically promoted tendon-to-bone healing by reducing inflammation and oxidative stress while enhancing stem cell chondrogenesis, fibrocartilage formation, and biomechanical strength (Chen B. et al., 2021). Molinaro et al. showed that in an *in vitro* human tenocyte model, tannic acid-coated curcumin-loaded acetalated dextran nanoparticles (TA-Cur-AcDEX NPs) developed via microfluidics showed strong anti-inflammatory and anti-fibrotic effects, significantly downregulating NF-κB, TGF-β, and MMP-3/9 expression while enhancing nanoparticle stability, uptake, and biocompatibility (Molinaro et al., 2023). Among endogenous antioxidant enzymes, PRDX5 has emerged as a crucial mitochondrial peroxidase that detoxifies hydrogen peroxide and alkyl hydroperoxides, particularly under mechanical strain. A study reported that PRDX5 is constitutively expressed in human tendon and significantly upregulated in degenerative rotator cuff tendon at both mRNA and protein levels. Immunohistochemistry and *in situ* hybridization localized PRDX5 predominantly to fibroblasts, with stronger expression in matrix-associated fibroblasts and endothelial cells in degenerate tendon, compared to weak expression in normal tendon (Wang et al., 2001; Yuan et al., 2004). The increased PRDX5 expression suggests activation of endogenous antioxidant defenses in response to elevated oxidative stress during tendon degeneration. On the other hand, melatonin, a pleiotropic indoleamine with strong antioxidant capacity, has gained attention for its ability to scavenge free radicals and upregulate antioxidant enzymes such as SOD and catalase (Zhang and Zhang, 2014). Melatonin also modulates mitochondrial dynamics by preserving membrane potential, inhibiting mPTP opening, and reducing cytochrome c release (Rahmani et al., 2024). In tendon models, melatonin treatment not only reduces oxidative markers but also promotes collagen alignment and enhances biomechanical recovery. Its safety profile, dual circadian and antioxidant regulatory properties make it a particularly attractive candidate for adjunctive therapy in both acute and chronic tendon injuries (Zhang J. et al., 2021; Kocadal et al., 2019). Kocadal et al. concluded that in a rat supraspinatus overuse tendinopathy model, exogenous melatonin administration (10 mg/kg twice daily) significantly reduced oxidative stress, iNOS and VEGF levels, improving the oxidant-antioxidant balance (lower OSI, TOS, and VEGF) and attenuating inflammation comparably to corticosteroid therapy (Kocadal et al., 2019). Together, these pharmacological antioxidants represent a diverse arsenal of therapeutics aimed at restoring redox homeostasis, reducing inflammation, and supporting tendon regeneration. Their efficacy, however, is highly dependent on dose, delivery method, and the phase of tendon healing, highlighting the need for targeted, temporally controlled strategies in clinical applications (Table 2).

**TABLE 2:** Pharmacological antioxidant agents targeting redox imbalance in tendon repair.

Category	Agent/Biomaterial	Mechanism of action	Key biological effects	Translational considerations
Small-Molecule antioxidants	N-acetylcysteine (NAC)	GSH precursor; direct ROS scavenger; activates integrin α5β1–PI3K/AKT signaling	Decreased ROS and apoptosis; increased COL1A1, SCX, TNMD; improved collagen alignment and tensile strength	Limited bioavailability; timing-dependent efficacy
	Vitamin C (ascorbic acid)	Cofactor for prolyl/lysyl hydroxylase; ROS neutralization	Increased collagen synthesis; decreased oxidative stress; improved tendon-bone healing	Rapid systemic clearance
	Vitamin E (α-tocopherol)	Lipid peroxidation inhibitor; membrane stabilization	Decreased COX-2 and iNOS; membrane protection	Poor tissue specificity
	Melatonin	Direct free radical scavenger; preserves mitochondrial membrane potential; ↑ SOD, CAT	Decreased OSI, TOS, and VEGF; improved collagen alignment	Favorable safety profile
	Curcumin	NF-κB/MAPK inhibition; NRF2 activation	Decreased inflammation; increased fibrocartilage formation; improved biomechanics	Low native bioavailability
	Resveratrol	SIRT1 activation; mitochondrial protection; NRF2 induction	Increased tenocyte proliferation; enhanced ECM organization	Requires delivery optimization
	Quercetin	NOX1/NOX4 inhibition; anti-inflammatory	Decreased ROS and IL-6; restored collagen I/III balance	Dose-dependent effects
Mitochondria-targeted antioxidants	MitoQ	Mitochondrial CoQ analog; neutralizes ETC-derived ROS	Preserves ATP synthesis; ↓ apoptosis; improved matrix integrity	Long-term dosing studies needed
	SS-31 (elamipretide)	Binds cardiolipin; stabilizes, ETC.,; reduces ROS leakage	Restored mitochondrial membrane potential; decreased MMP1 and FABP4; improved tendon strength	Clinical tendon data limited

In addition to pharmacological antioxidant strategies, autologous biologic therapies have also emerged as relevant adjuncts in redox-oriented tendon regeneration, particularly because they combine regenerative signaling with immunomodulatory and, in some settings, antioxidant effects (Koshy et al., 2025). Platelet-rich plasma (PRP), the most widely studied autologous biologic, has been shown to enhance tenocyte proliferation, matrix gene expression, and tendon repair responses (Nishio et al., 2020; Hudgens et al., 2016). Tognoloni et al. investigated the activation of the Nrf2 antioxidant signaling pathway in tenocytes under oxidative stress and evaluated the effects of PRP, indicated that PRP promotes Nrf2 nuclear translocation, enhances antioxidant enzyme expression, and reduces oxidative damage in tendon cells (Tognoloni et al., 2023). However, PRP effects appear to be formulation-dependent, as some preparations, particularly those enriched with leukocytes, may also trigger transient inflammatory and oxidative signaling in tendon fibroblasts, which may partly explain the heterogeneity of clinical outcomes (Lansdown and Fortier, 2017). In parallel, bone marrow aspirate concentrate (BMAC) and autologous adipose-derived stromal/stem cell therapies provide progenitor cells together with trophic factors that can suppress inflammation, support neovascularization, and improve matrix remodeling (Pintore et al., 2023; Veronesi et al., 2015; Kokubu et al., 2020). Preclinical studies further suggest that these cell-based autologous strategies may mitigate oxidative injury indirectly through paracrine regulation of redox-sensitive pathways and enhancement of the local reparative microenvironment (Imam et al., 2017; Kim et al., 2017). Nevertheless, despite their promise, autologous therapies remain limited by donor-to-donor variability, inconsistent preparation protocols, uncertain dose composition, and the need for more standardized mechanistic studies linking their clinical benefit to specific ROS-modulating effects.

### 8.2 Antioxidant-functionalized biomaterials in tendon repair

Biomaterial-based interventions have evolved as promising adjuncts in tendon repair, for their structural support, and also as platforms for localized, controlled antioxidant delivery. Given the critical role of oxidative stress in tendon degeneration, delayed healing, and fibrotic remodeling, functionalizing scaffolds with antioxidant properties has emerged as a rational strategy to modulate the redox microenvironment, enhance cellular survival, and promote regenerative outcomes. These biomaterials offer spatiotemporal control over antioxidant release and can be tailored to mimic the mechanical and biochemical properties of native tendon ECM (Table 3). Biodegradable polymers such as gelatin, collagen, chitosan, silk fibroin, and polycaprolactone (PCL) have been extensively used as scaffolds for tendon tissue engineering (Alhaskawi et al., 2024; Yarahmadi et al., 2024). When functionalized with antioxidants such as curcumin, quercetin, resveratrol, or ascorbic acid, these scaffolds significantly reduce intracellular ROS levels in tenocytes and TSPCs exposed to oxidative stress. For example, curcumin-loaded electrospun PCL scaffolds have demonstrated sustained antioxidant release, reduced expression of pro-inflammatory cytokines, and enhanced collagen type I deposition in preclinical tendon defect models (Yoshikawa et al., 2025; Sadeghianmaryan et al., 2020). Similarly, resveratrol-incorporated collagen hydrogels not only scavenge ROS but also activate SIRT1 and NRF2 signaling pathways, promoting matrix organization and tenocyte proliferation (Ciccone et al., 2022; Freedman et al., 2022). Zhu et al. designed single-atom artificial antioxidases (AAOs) with Fe-, Co-, and Ni-N_4_ centers, which efficiently scavenge ROS *via* spin polarization-enhanced catalysis; notably, Fe-AAO showed superior catalase-like and Co-AAO strong peroxidase-like activity, together reducing oxidative stress and accelerating tendon regeneration (Zhu et al., 2024). Crucially, they validated antioxidant efficacy *in vivo* in a rat tendon injury model using local delivery, demonstrating real tissue-level protection. Yao et al. found that in a rat Achilles tendon injury model, biomimetic multilayer polycaprolactone/sodium alginate (PCL/ALG) hydrogel scaffolds loaded with melatonin significantly enhanced tendon regeneration by promoting tenocyte proliferation, collagen I and decorin expression, and ECM organization while activating the NRF2/HO-1 antioxidant pathway and reducing ROS production and macrophage infiltration (Yao et al., 2022). Furthermore, an advanced strategy involves the design of redox-responsive or ROS-degradable biomaterials, which can sense oxidative cues in the microenvironment and respond dynamically. These smart materials incorporate thioketal linkers, boronate esters, or ROS-cleavable micelles, which degrade specifically in the presence of superoxide or hydrogen peroxide (Rinaldi et al., 2022; Zhou et al., 2023). Upon degradation, they release encapsulated antioxidants or therapeutic agents directly at the injury site. In addition, nanotechnology has enabled the development of antioxidant-loaded nanoparticles, which can be embedded within hydrogels, electrospun fibers, or injectable carriers. These nanoparticles can be further surface-modified with collagen-binding peptides, hyaluronic acid, or RGD motifs to enhance tendon-specific targeting and cell-scaffold interactions (Daré and Lautenschlager, 2025). Wang et al. study found that delivering miR-494-3p *via* a PLGA nanoparticle-hydrogel system significantly enhanced Achilles tendon healing in rats by sustainedly releasing miR-494-3p into tenocytes, where it suppressed CXXC4 translation, activated collagen I synthesis, improved biomechanical strength, and reduced inflammation (Wang G. H. et al., 2025). Adjei-Sowah et al. described a tendon-targeted nanoparticle (NP) delivery system functionalized with a TRAP-binding peptide (TBP) to enhance accumulation in injured tendon. In a flexor digitorum longus transection and repair mouse model, TBP-conjugated NPs loaded with the anti-fibrotic drug Niclosamide were administered systemically 7 days post-injury. Compared with saline and free-drug controls, TBP-NP-treated mice demonstrated significantly improved range of motion and enhanced mechanical properties, confirming effective tendon-specific targeting and functional repair (Adjei-Sowah et al., 2025). Faustini et al. developed bioreducible poly (amidoamine) nanoparticles to locally deliver chemically modified mRNAs encoding IL1RA and PDGF-BB into a rat patellar tendon defect model. *In vitro*, PDGF-BB cmRNA enhanced tendon cell proliferation and migration, while IL1RA cmRNA reduced pro-inflammatory cytokine expression. *In vivo* delivery at day 7 post-injury improved collagen fiber alignment, reduced early inflammation (COX-2, CD68), limited late fibrosis (S100a4), and enhanced overall tendon repair, supporting a dual anti-inflammatory and pro-regenerative strategy. (Faustini et al., 2025).

**TABLE 3:** Antioxidant-functionalized biomaterials and redox-responsive delivery platforms for tendon regeneration.

Category	Biomaterial platform	Redox strategy	Key biological outcomes	Advantages
Electrospun scaffolds	Curcumin- or resveratrol-loaded PCL/PLA fibers	Sustained antioxidant release	Decreased IL-1β and TNF-α levels; increased collagen I deposition; improved mechanical strength	ECM-mimetic architecture
Hydrogels (melatonin-loaded PCL/ALG)	NRF2/HO-1 activation; reduction of reactive oxygen species	Decreased macrophage infiltration; enhanced extracellular matrix organization	Injectable; spatiotemporal control	Degradation kinetics optimization
Artificial antioxidases (Fe-, Co-, Ni-N4 AAOs)	Catalase- and peroxidase-like reactive oxygen species scavenging	Accelerated tendon regeneration; decreased oxidative stress markers	Enzyme-mimetic catalytic activity	Long-term biosafety
Redox-responsive scaffolds (thioketal/boronate linkers)	Reactive oxygen species-triggered degradation and controlled release	Targeted antioxidant delivery at the injury site	Smart, stimulus-responsive	Manufacturing complexity
Nanoparticle systems (PLGA, TBP-functionalized NPs)	Targeted delivery of anti-fibrotic drugs or microRNA	Reduced fibrosis; improved biomechanical recovery	Systemic or localized targeting	Regulatory pathway complexity
Gene-activated scaffolds (cmRNA nanoparticles)	PDGF-BB and IL1RA expression	Reduced inflammation; improved collagen alignment	Dual regenerative and anti-inflammatory effect	Stability and immune response

Electrospinning technology has been widely used to fabricate nanofibrous scaffolds that replicate the architecture of native tendon ECM. Functionalization of electrospun fibers with antioxidants enhances both mechanical integrity and biochemical modulation (Xue et al., 2019). For instance, core-shell electrospun fibers with antioxidant-loaded cores and bioadhesive outer shells allow sequential release; initial cell attachment followed by ROS regulation (Li et al., 2025b). Uyanik et al. indicated that electrospun bioabsorbable PLGA nanofiber membranes significantly reduced peritendinous adhesions in a rat Achilles tendon injury model without impairing biomechanical strength, indicating strong potential for clinical use in tendon repair (Uyanik et al., 2022). Di Marco et al. engineered aligned electrospun PLA scaffolds coated with type I collagen and loaded with the antifibrotic drug Rolipram to create a tendon-mimetic drug delivery system. Collagen coating enhanced mechanical strength (Young’s modulus increased from 103 to 140 MPa in dry conditions) and significantly slowed Rolipram release, following Fickian diffusion kinetics. *In vitro*, collagen-coated drug-loaded scaffolds supported human tenocyte adhesion, preserved TNMD expression, and increased type I collagen deposition after 14 days, indicating maintenance of tenogenic phenotype and antifibrotic potential (Di Marco et al., 2025). Jiang et al. developed a bidirectional hydrated fibrous gene patch (siRNA@DHP-PB) for preventing peritendinous adhesions. The outer self-healing hydrogel layer delivers TGF-β1 siRNA/TAT in a pH/GSH-responsive manner to suppress early fibrotic signaling, while the inner PLCL electrospun membrane releases berberine to provide antioxidant, anti-inflammatory, and antibacterial effects. In a rat Achilles adhesion model, the patch significantly reduced ROS levels, inflammatory markers (TNF-α), Col-3 deposition, and TGF-β1 expression, resulting in decreased fibrosis and improved functional recovery (Jiang et al., 2025). Nevertheless, the co-application of TSPCs or MSCs with antioxidant-functionalized scaffolds enhances the regenerative potential of these constructs. Zhao et al. developed a tannic acid-modified decellularized tendon scaffold (DTS-TA) that enhances tendon regeneration by combining antioxidant and anti-inflammatory properties; the scaffold effectively scavenges excessive ROS, promotes M2 macrophage polarization, reduces IL-6 and IL-1β, and increases IL-4 expression, while also improving mechanical strength, hydrophilicity, and biocompatibility (Zhao et al., 2024). Rieber et al. developed an electrospun DegraPol tube delivering secretome from a co-culture of adipose-derived stem cells and tenocytes for Achilles tendon repair in rabbits, showing that this cell-free implant significantly reduced adhesion formation (−22%), lowered tendon swelling (−32% cross-sectional area), and restored near-native load and stiffness, demonstrating its strong potential as a biodegradable, secretome-releasing scaffold to enhance tendon healing and biomechanics (Rieber et al., 2025). Furthermore, Datla et al. demonstrated that cyclic mechanical stimulation (5% strain, 0.25–0.5 Hz) of PCL/tdECM electrospun membranes in a bioreactor enhanced tenogenic differentiation of MSCs, promoted collagen organization, and mitigated ROS generation through mechanotransduction-driven FAK-Smad3 signaling, yielding a biomimetic platform for tendon repair (Datla et al., 2025).

## 9 Future directions and challenges

Although substantial progress has been made in elucidating the molecular interplay between ROS, mitochondrial dynamics, calcium signaling, and antioxidant defense systems in tendon biology, several conceptual and translational challenges remain. A central limitation lies in the incomplete understanding of the spatiotemporal dynamics of ROS generation *in vivo*. Tendon tissue is structurally heterogeneous, with distinct mechanical, metabolic, and vascular characteristics across the midsubstance, enthesis, and peritendinous regions. Emerging redox biology research indicates that ROS signaling is highly compartmentalized, with mitochondrial, cytosolic, and membrane-associated pools exerting distinct functional roles (Lennicke and Cochemé, 2021; Hong et al., 2024). However, most current tendon studies rely on bulk oxidative stress markers rather than real-time or compartment-specific measurements. Future investigations should incorporate genetically encoded redox biosensors, high-resolution intravital imaging, and single-cell transcriptomic analyses to map localized redox gradients during tendon degeneration and repair. Such approaches will clarify whether tendinopathy reflects generalized oxidative overload or a failure of localized antioxidant buffering within specific cellular niches. Nevertheless, many therapeutic strategies focus primarily on ROS scavenging rather than restoring upstream mitochondrial homeostasis. Increasing evidence indicates that dysregulation of mitochondrial quality control, including impaired mitophagy (PINK1/Parkin axis), altered fission-fusion balance (DRP1, MFN2, OPA1), and destabilized mitochondrial-ER contact sites, contributes to sustained ROS leakage and defective bioenergetics (Cheng et al., 2024; Kračun et al., 2025). Selective targeting of NADPH oxidases (NOX enzymes) represents another promising yet complex direction. NOX1 and NOX4 are increasingly implicated in tendon inflammation, diabetic degeneration, and fibrotic remodeling, yet these enzymes also participate in physiological mechanotransduction and adaptive redox signaling (Ackerman et al., 2021; Begum et al., 2022). The challenge lies in achieving isoform-specific and temporally controlled inhibition without suppressing beneficial ROS-dependent repair mechanisms. Advanced strategies such as tendon-targeted nanoparticles, redox-responsive biomaterials, or localized delivery systems capable of transient NOX modulation may offer improved therapeutic precision. Determining the optimal timing of NOX inhibition across the inflammatory, proliferative, and remodeling phases of healing will be essential to avoid impairing regenerative cascades. In addition, redox regulation of TSPC fate constitutes another emerging Frontier. Accumulating evidence suggests that ROS levels influence lineage commitment, shifting differentiation toward tenogenic, adipogenic, or fibrotic phenotypes depending on oxidative tone. However, the integration of redox signaling with transcriptional regulators such as SCX, Mohawk (MKX), YAP/TAZ, and HIF-1α remains incompletely characterized (Dai et al., 2019; Zhang et al., 2026). Moreover, epigenetic modifications, including redox-sensitive DNA methylation and histone acetylation, may play a pivotal role in sustaining maladaptive tendon remodeling (Cyr and Domann, 2011; Hatzinger et al., 2026). Future studies integrating single-cell epigenomics, metabolic flux analysis, and mechanobiology will be required to clarify how redox states coordinate stem cell plasticity and tissue regeneration. Understanding this interface may enable the development of redox-guided regenerative strategies that optimize TSPC function without promoting fibrosis. Furthermore, translational challenges also persist in antioxidant therapy. While agents such as N-acetylcysteine, mitochondrial-targeted antioxidants (MitoQ, SS-31), and NOX inhibitors have demonstrated efficacy in preclinical models, clinical translation remains limited by poor bioavailability, nonspecific distribution, and insufficient phase-specific targeting (Sakai and Kumagai, 2025; Wang S. et al., 2025). Emerging antioxidant-functionalized biomaterials and redox-responsive scaffolds represent promising platforms capable of delivering controlled, localized therapy within the mechanically dynamic tendon microenvironment (Sakai and Kumagai, 2025; Wang S. et al., 2025). Nevertheless, long-term safety, degradation kinetics, and immunomodulatory effects require further evaluation before clinical implementation. Furthermore, the dual nature of ROS, as both signaling mediators and pathological agents, necessitates precise dose optimization to avoid over-suppression of physiological redox processes.

Finally, systemic metabolic conditions such as diabetes, aging, and obesity introduce additional complexity by altering baseline oxidative tone, mitochondrial resilience, and inflammatory sensitivity. Personalized redox profiling may therefore become necessary to tailor therapeutic strategies based on patient-specific metabolic status. Integration of redox biomarkers, advanced imaging modalities, and biomechanical assessment could facilitate precision medicine approaches in tendon injury management.

## 10 Conclusion

Understanding the redox landscape of tendon biology reveals how finely tuned oxidative and antioxidant responses influence tissue integrity and healing. Disruption of this balance, whether through excessive ROS production or insufficient antioxidant activity, contributes to pathological remodeling and impaired regeneration. Targeting redox-sensitive pathways offers a promising direction for developing more effective, mechanism-based therapies for tendon injury.
